# Metabolome Profiling Supports the Key Role of the Spike in Wheat Yield Performance

**DOI:** 10.3390/cells9041025

**Published:** 2020-04-21

**Authors:** Omar Vergara-Diaz, Thomas Vatter, Rubén Vicente, Toshihiro Obata, Maria Teresa Nieto-Taladriz, Nieves Aparicio, Shawn Carlisle Kefauver, Alisdair Fernie, José Luis Araus

**Affiliations:** 1 Integrative Crop Ecophysiology Group, Plant Physiology Section, Faculty of Biology, University of Barcelona, Diagonal 643, 08028 Barcelona, Spain; omarvergaradiaz@gmail.com (O.V.-D.); thomasva@gmx.de (T.V.); vicente@mpimp-golm.mpg.de (R.V.); sckefauver@ub.edu (S.C.K.); 2 AGROTECNIO (Center of Research in Agrotechnology), 25198 Lleida, Spain; 3 Max Planck Institute of Molecular Plant Physiology, Am Mühlenberg 1, 14476 Potsdam, Germany; tobata2@unl.edu (T.O.); Fernie@mpimp-golm.mpg.de (A.F.); 4 National Institute for Agricultural and Food Research and Technology (INIA), Ctra de la Coruña 7.5, 28040 Madrid, Spain; mtnieto@inia.es; 5 Technological and Agrarian Institute of Castilla y León (ITACyL), Agricultural Research. Ctra Burgos km 119, 47041 Valladolid, Spain; ApaGutNi@itacyl.es

**Keywords:** LASSO regression, leaf, metabolome, spike, water stress, grain yield, wheat

## Abstract

Although the relevance of spike bracts in stress acclimation and contribution to wheat yield was recently revealed, the metabolome of this organ and its response to water stress is still unknown. The metabolite profiles of flag leaves, glumes and lemmas were characterized under contrasting field water regimes in five durum wheat cultivars. Water conditions during growth were characterized through spectral vegetation indices, canopy temperature and isotope composition. Spike bracts exhibited better coordination of carbon and nitrogen metabolisms than the flag leaves in terms of photorespiration, nitrogen assimilation and respiration paths. This coordination facilitated an accumulation of organic and amino acids in spike bracts, especially under water stress. The metabolomic response to water stress also involved an accumulation of antioxidant and drought tolerance related sugars, particularly in the spikes. Furthermore, certain cell wall, respiratory and protective metabolites were associated with genotypic outperformance and yield stability. In addition, grain yield was strongly predicted by leaf and spike bracts metabolomes independently. This study supports the role of the spike as a key organ during wheat grain filling, particularly under stress conditions and provides relevant information to explore new ways to improve wheat productivity including potential biomarkers for yield prediction.

## 1. Introduction

The projections of the effects of global change, including increases in temperature and dryness in many regions, threaten crop production in the coming years [1,2]. Wheat is particularly sensitive to drought at the flowering and grain filling stages, while more than 50% of the land under wheat cultivation is already affected by periodic droughts [3]. Durum wheat (*Triticum turgidum* L. subsp. *durum* (Desf) Husn.) is the primary crop in the south and east of the Mediterranean basin with the European Union being the leading global producer [4]. However, genetic gain for grain yield in post-green revolution wheats has stagnated or even declined in some regions [5]. In this sense, wheat breeding for water stress tolerance is urgent, particularly in regions that are highly sensitive to global climate change such as the Mediterranean Basin [2]. Strategies to combat these challenges should involve enhancing stress resilience and progressing field-based high-throughput phenotyping.

Multi-omics techniques have recently been used for the characterization of wheat performance under water stress [6,7]. These are crucial to understand the underlying molecular mechanisms of plant responses that confer genotypic resilience to stress conditions. Water stress is known to trigger major reprogramming of plant metabolism including a decrease in photosynthetic carbon metabolism via restriction in CO_2_ diffusion and inhibition of CO_2_ assimilation [8], whereas photorespiration and the accumulation of reactive oxygen species increases [9]. Furthermore, water stress generally inhibits nitrogen (N) assimilation and stimulates amino acid catabolism, while the accumulation of certain amino acids can function as osmoprotectants or signaling molecules [10,11,12]. For instance, an accumulation has long been documented for certain amino acids such as proline, γ-aminobutyric acid (GABA) and isoleucine in wheat leaves in response to water stress [6,13,14]. However, no previous evidence for a functional role of these metabolites has been reported in spike bracts.

In the last few years, far more attention has been paid to the role of photosynthetic organs other than the leaf blades in plant stress acclimation, with the spikes being particularly relevant. Indeed, the spike has been shown to be a major contributor of photosynthates [15]. Some studies have revealed that the contribution of wheat bracts to grain filling is considerable due to their higher and/or more steady photosynthesis, high carbon refixation rates, delayed senescence and high water-use efficiency compared to the flag leaves, particularly under water stress conditions [7,16,17,18,19]. In addition, an up-regulation of nitrogen and respiration metabolism has been reported in wheat spikes under water stress, revealing that spike bracts are active sites for nitrogen assimilation [7]. In sharp contrast, the metabolic and transcriptomic profiles of leaves and roots in cereals have been used for predicting complex agronomic traits including yield [20,21,22,23,24]. However, the metabolome of wheat spike bracts and their response to water stress has not been characterized and, in fact, the metabolic role of the spike is not yet understood to any depth. In the present work we aimed to (i) characterize the metabolome of the flag leaf and spike bracts -specifically the spike glumes and lemmas- in durum wheat in response to water stress, (ii) explore the existence of organ-specific metabolic traits and physiological functions, (iii) identify metabolites associated with genotypic outperformance, and (iv) model metabolite-grain yield association.

## 2. Materials and Methods

### 2.1. Plant Material and Experimental Set Up

Field trials were carried out during the 2014/15 growing season at three locations with different growth conditions in Spain. Agronomic information together with weather, irrigation and soil information are detailed in Table 1.

The study is based on five modern (i.e., semidwarf) commercial varieties of durum wheat (*Triticum turgidum* L. subsp *durum* (Desf) Husn.) released during the last thirty years in Spain. The varieties Sula, Dorondon, Pelayo, Don Sebastian and Kiko Nick, registered in the years 1994, 1999, 2003, 2004 and 2009, respectively, were selected from a panel of 20 semi-dwarf post-Green-Revolution varieties as they have been demonstrated to be representative of yield performance variability under water stress conditions while neither phenology differences—days to heading—nor genotype per environment interaction were shown [5] (Figure S1). Information regarding each cultivar’s pedigree, characteristics and breeders is provided in Table S1. These five cultivars derive from two main breeding sources: (i) CIMMYT (the International Maize and Wheat Improvement Center) lines, developed under the irrigation conditions of NW Mexico but aimed at wide adaptation, and (ii) lines derived from European (mostly Italian) germplasm. Sula is included in the first category, along with Don Sebastian. While the former genotype is an established CIMMYT cultivar, the latter is an advanced line from CIMMYT that was further selected as a cultivar based in its adaptation to the warm conditions of Andalusia (South Spain). Within the second category Pelayo derives from Italian germplasm, and Dorondon has been targeted towards the inland (i.e., continental) agrosystems of the Iberian Peninsula, with both cultivars having been bred in Spain. Finally, Kiko Nick is the result of crossing germplasm from CIMMYT and Italy, but was bred in Spain and selected for wide adaptation.

**Figure 1:**
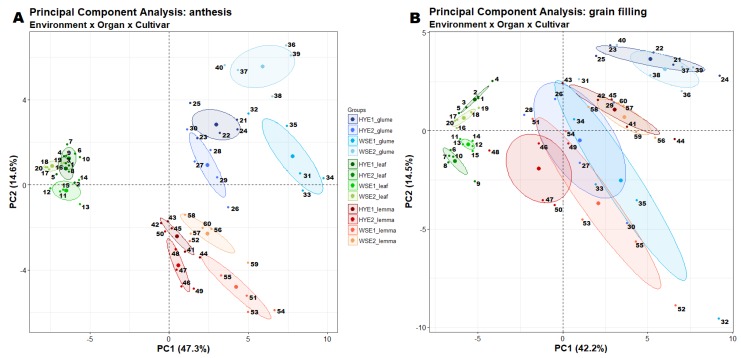
Principal component analysis of metabolite profiles of leaves, glumes and lemmas at the anthesis (**A**) and grain filling stages (**B**). Individuals are numbered in the graph and labeled in the legend by the five cultivars: DO, Dorondon; DS, Don Sebastian; KN, Kiko Nick; PE, Pelayo; SU, Sula; and by the four environments: HYE1 and HYE2, high-yielding environments 1 and 2 (corresponding to the irrigated trial of Zamadueñas and to El Majano trial, respectively); WSE1 and WSE2, water stress environments 1 and 2 (corresponding to the rainfed trials of Aranjuez and Zamadueñas, respectively). 1, HYE1 leaf KN; 2, HYE1 leaf PE; 3, HYE1 leaf DO; 4, HYE1 leaf DS; 5, HYE1 leaf SU; 6, HYE2 leaf KN; 7, HYE2 leaf PE; 8, HYE2 leaf DO; 9, HYE2 leaf DS; 10, HYE2 leaf SU; 11, WS1 leaf KN; 12, WS1 leaf PE; 13, WS1 leaf DO; 14, WS1 leaf DS; 15, WS1 leaf SU; 16, WS2 leaf KN; 17, WS2 leaf PE; 18, WS2 leaf DO; 19, WS2 leaf DS; 20, WS2 leaf SU; 21, HYE1 glume KN; 22, HYE1 glume PE; 23, HYE1 glume DO; 24, HYE1 glume DS; 25, HYE1 glume SU; 26, HYE2 glume KN; 27, HYE2 glume PE; 28, HYE2 glume DO; 29, HYE2 glume DS; 30, HYE2 glume SU; 31, WS1 glume KN; 32, WS1 glume PE; 33, WS1 glume DO; 34, WS1 glume DS; 35, WS1 glume SU; 36, WS2 glume KN; 37, WS2 glume PE; 38, WS2 glume DO; 39, WS2 glume DS; 40, WS2 glume SU; 41, HYE1 lemma KN; 42, HYE1 lemma PE; 43, HYE1 lemma DO; 44, HYE1 lemma DS; 45, HYE1 lemma SU; 46, HYE2 lemma KN; 47, HYE2 lemma PE; 48, HYE2 lemma DO; 49, HYE2 lemma DS; 50, HYE2 lemma SU; 51, WS1 lemma KN; 52, WS1 lemma PE; 53, WS1 lemma DO; 54, WS1 lemma DS; 55, WS1 lemma SU; 56, WS2 lemma KN; 57, WS2 lemma PE; 58, WS2 lemma DO; 59, WS2 lemma DS; 60, WS2 lemma SU.

For each trial, plants were sown in a randomized block design with three replicates and four growing conditions were considered. First, two rainfed trials in Colmenar de Oreja and Zamadueñas that are highly restrictive environments were grouped as water stress (WS) conditions. Second, the supplemental irrigation trial of Zamadueñas and the rainfed trial of El Majano which is characterized as a high-yielding environment due to its closeness to the Guadalquivir River (i.e., high-water table level) were hereafter considered as high-yielding (HY) conditions. Trial grouping was further supported by yield and carbon isotope data of the same plant material in retrospective studies [5,25] as well as by the similarities in metabolite variability (Figure 1) including those between the rainfed and irrigated trials of Zamadueñas (Table S2).

Plant height (PH) was measured at grain filling. At harvest, grains were dried in an oven at 60 °C for 48 h to steady weight and grain yield (GY) was determined by harvesting the whole plot. In addition, total biomass was measured and the number of grains spike^−1^ was counted in a set of 10 plants per plot. Then, thousand kernel weight (TKW) and harvest index (HI) were calculated.

### 2.2. Spectral and Thermal Field Measurements

The flag leaf, spike and canopy spectral signatures were measured around midday on sunny days with a FieldSpec4 (ASD Inc. PANalytical Company, Boulder, CO, USA) full-range portable spectroradiometer. The reflectance spectra of three flag leaves and three spikes were recorded for each plot with an ASD leaf clip accessory. Canopy spectra were measured with a pistol grip coupled by an optical fiber to the FieldSpec4 spectrometer. Measurements were made one meter above the plot canopy in a zenithal plane and the reflectance was calibrated every 15–20 min with a Spectralon white reference panel. Spectra were acquired at the crop development stages of anthesis and grain filling, which are 69 and 74 in the Zadoks scale [26] respectively, on 13 April and 11 May in El Majano, 12 and 25 May in Colmenar de Oreja and 15 and 28 May in Zamadueñas. Then, three water-related spectral reflectance indices were calculated: the Normalized Difference Water Index (NDWI) [27], the Normalized Water Index (NWI) [28] and the Normalized Difference Moisture Index (NDMI) [29].

Canopy temperature was acquired in the afternoon (about 2PM solar time) from a remotely piloted aircraft system flown in clear sky conditions using a Mikrokopter Oktokopter 6S12 XL eight rotor UAV (HiSystems GmbH, Moomerland, Germany) at an above ground level altitude of 50 m. A FLIR Tau2 640 (FLIR Systems, Nashua, NH, USA) thermal camera was mounted on a MK HiSight SLR2 camera platform and programmed for continuous image capture with an image acquisition rate of 20 s^−1^, a resolution of 640 × 520 pixels and an estimated ground spatial resolution of 54 mm per pixel. Thermal images were exported using the TEAX ThermoViewer v1.3.12 and converted to 32bit temperature in Celsius using a custom batch processing macro function in FIJI software [30].

### 2.3. Leaf and Spike Metabolite Profiling and Isotope Analyses

Three flag leaf blades and three spikes per plot were harvested and immediately frozen in dry ice at the stages of anthesis and middle grain filling on the same dates mentioned before. All glumes and lemmas of each of the three collected spikes per replicate were separated, and the three partitioned organs were ground in liquid nitrogen. Although awns can be important photosynthetic bracts contributing to yield [31] they are more affected by early senescence and were discarded in the current work. One hundred milligrams of fresh material powder from 360 samples were used for gas chromatography-mass spectrometry (GC-MS).

Metabolite extraction and derivatization were performed as an adaptation of the procedure described in [32,33]. One microliter of each sample solution was injected into a gas chromatography time-of-flight mass spectrometry (GC–TOF–MS) system (Pegasus III, Leco, St Joseph, MO, USA). The chromatogram was evaluated using GC–TOF–MS ChromaTOF software (Pegasus, LECO, St Joseph, MO, USA). Peaks in the chromatograms were manually annotated and ion intensity was determined using TagFinder software [34] and with a reference library derived from the Golm Metabolome-Database for compound identification [35].

The stable carbon (^13^C:^12^C) isotope ratio as well as the nitrogen concentration (%N) were measured in the flag leaves at grain filling and in mature grain dry matter using an elemental analyzer (Flash 1112 EA; Thermo Finnigan, Bremen, Germany) coupled with an isotope ratio mass spectrometer (Delta C IRMS, Thermo Finnigan) operating in a continuous flow mode. Samples of 0.7–1 mg of leaf and grain dry matter from each plot, together with reference materials, were weighed and sealed into tin capsules. Measurements were conducted at the Scientific Facilities of the University of Barcelona. Isotopic values were expressed in composition notation (δ) as follows: δ^13^C (‰) = [(^13^C/^12^C)_sample_/(^13^C/^12^C)_standard_] − 1, where ‘sample’ refers to plant material and ‘standard’ to international secondary standards of known ^13^C:^12^C ratios (IAEA CH7 polyethylene foil, IAEA CH6 sucrose, and USGS 40 L-glutamic acid) calibrated against Vienna Pee Dee Belemnite calcium carbonate with an analytical precision (standard deviation) of 0.10‰. Grain nitrogen yield (GNY) was then calculated as the product of GY and grain %N per plot.

### 2.4. Statistical Analysis

R 3.5.1 [36] was used for conducting multivariate ANOVAs and heatmap analyses were undertaken with the GPLOTS package [37]. Principal component analysis (PCA) and correlation network analyses were performed with the PCA3D and QGRAPH packages, respectively [38,39]. Figures were drawn with SigmaPlot 10.0 (Systat Software Inc., San Jose, CA, USA).

The metabolite data used for the prediction models of GY were first revised so that those metabolites with more than 10% missing data over all samples were removed from the dataset. The remaining missing values were then interpolated with the DMwR package [40] with the K-Nearest Neighbor Classification method using RStudio 3.2.2.

Yield prediction models were performed with flag leaves and spike bracts metabolite profiles at the anthesis and grain filling stages and by considering an additional log_2_-transformation of metabolite intensities. Individual (i.e., single plot) metabolic data for the HY and WS conditions were combined into one dataset. Identification of metabolite GY associations and GY prediction based on metabolite data was performed using least absolute shrinkage and selection operator (LASSO) regression. LASSO is a penalized logistic regression that can handle large number of predictors, which are in turn highly collinear, and precise variable selection and prediction despite small sample size. Analysis was carried out with SAS software 9.4 (SAS Institute Inc., Cary, NC, USA) applying the *proc glmselect* procedure. To increase the robustness of the results, five-fold cross-validation (CV) was conducted. In total 100 CV runs (20 times five-fold CV) were performed. For these, 100 subsets were extracted from the full dataset. Each of the subsets comprised 75% of the data points and was randomly selected. The subsets were taken as training sets for the identification of metabolite grain yield associations and for the estimation of their effects. The remaining 25% of the data was used as a validation set. To estimate the proportion of variance in grain yield explained by the model, the unbiased estimator Adj-R^2^ [41] was calculated for each subset. As a measure of accuracy, the root mean square error (RMSE) was calculated. Effects for each metabolite were extracted as a regression coefficient of the respective metabolite directly from the LASSO model. In addition, the count of each metabolite over all training sets was recorded and referred to as the detection rate. This value was taken as a measure of importance of the specific metabolite grain yield association. To determine the predictive ability of the full model for grain yield, the regression estimates, obtained using the training sets, were used to predict the grain yield value of the remaining 25% of data forming the validation sets. The predictive ability was defined to be the squared Pearson product-moment correlation between predicted and observed phenotypic values. The statistics provided for each model (R^2^, Adj-R^2^, RMSE, and metabolite effect for the training and validation sets) were averaged across all 100 CV runs to obtain the results.

In addition to the predictive ability that was estimated based on the validation dataset by combining data of HY and WS conditions, the predictive ability of the models was tested in HY and WS conditions separately. For this, multiple regression was performed by setting the metabolites that showed a detection rate of at least 70% according to the LASSO variable selection.

## 3. Results

All the agronomic traits studied were significantly lower under water WS compared to the HY conditions (Table 2). Specifically, the largest reductions were detected in grain yield (GY, 37.3%), grain nitrogen yield (GNY, 27.0%) and biomass (24.6%) followed by reductions in thousand kernel weight (TKW, 19.1%), harvest index (HI, 13.8%) and the number of grains spike^−1^ (10.3%). In addition, genotypic differences were detected in GY, TKW and in the number of grains spike^−1^. GY was significantly higher in Pelayo and Sula, followed by Kiko Nick and Dorondon with intermediate GYs, and, lastly, Don Sebastian showed the lowest grain yield. TKW was higher in Don Sebastian, Pelayo and Kiko Nick and lower in Sula and Dorondon. The number of grains spike^−1^ was higher in Dorondon and Sula, intermediate in Pelayo and lower in Kiko Nick and Don Sebastian. Grain nitrogen content was significantly higher under WS conditions, whereas it was significantly lower in the genotypes Dorondon and Sula, intermediate in Kiko Nick and Pelayo and higher in Don Sebastian.

At both growth stages canopy temperature was significantly higher under WS conditions (Table 3). The three spectral reflectance indices selected for the assessment of canopy, leaf and spike water status (NDMI, NDWI and NWI) showed a significantly higher water signal under HY conditions compared to WS conditions at both growth stages. Both leaf and grain δ ^13^C were significantly higher under WS conditions. No significant genotype per environment interaction was obtained for any of these agronomic or phenotypic traits.

### 3.1. Metabolome Differences between Organs and Growth Stages

Marked differences in the metabolite profiles were in part attributed to inter-organ and growth stage variability. At anthesis, the PCA (Figure 1) explained up to 61.9% of metabolome variance with samples being grouped according to the organ and growing environment. The separation between spike bracts and environments was less clear at grain filling. Several environments per organ and/or stage interactions were significant because metabolic changes were closely associated with the organ whereas many metabolites showed differential increasing or decreasing phenological patterns.

**Figure 2:**
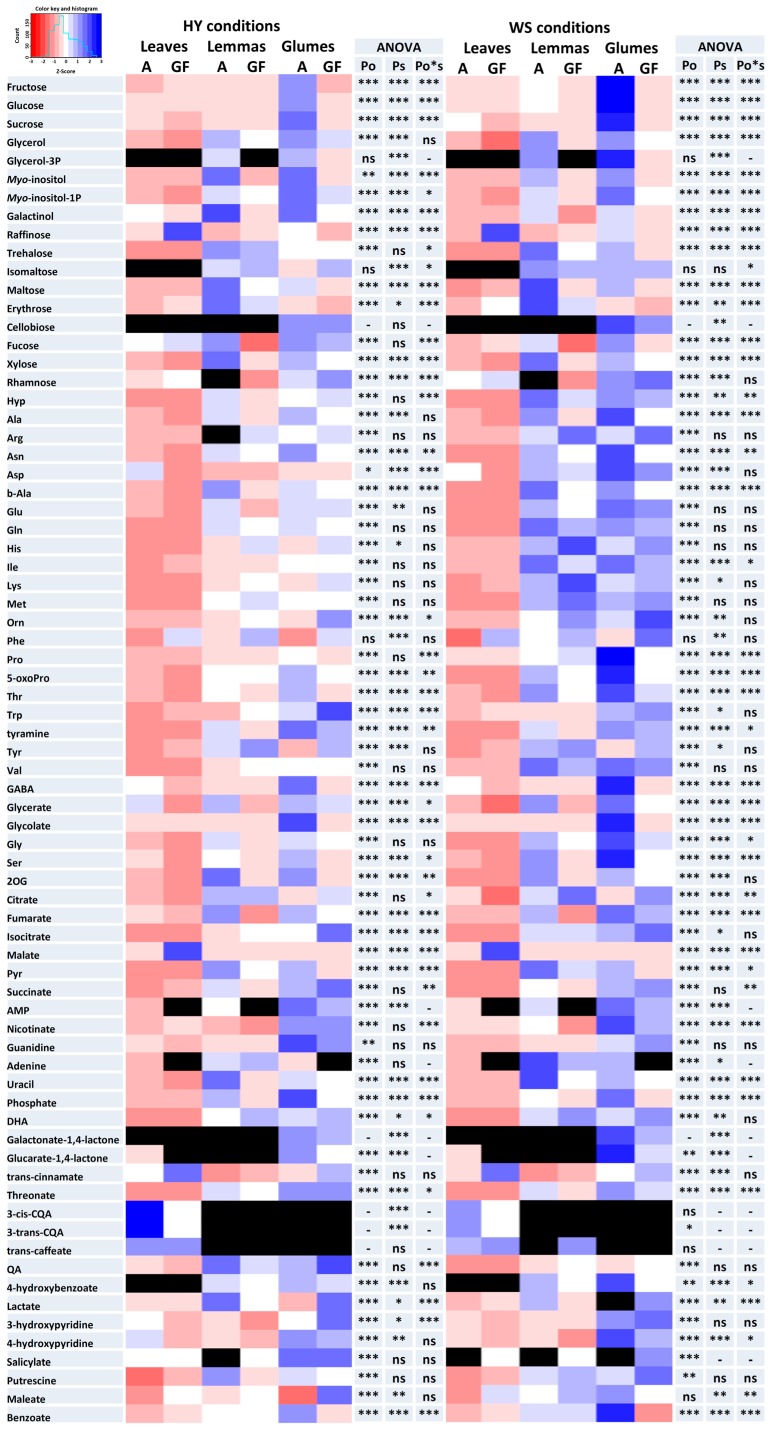
Heatmap of the metabolite profiles of leaves, lemmas and glumes at the anthesis (A) and grain filling (GF) stages under high-yielding (HY) and water stress (WS) conditions. *p*-values are shown for the organ (P_o_) and stage (P_s_) factors and their interaction (P_o*s_). The red-blue color scale was obtained from Z-score transformation of actual values. Black indicates metabolites that were not detected. *, *p* < 0.05; **, *p* < 0.01; ***, *p* < 0.001.

Except for aspartate (Asp), malate, raffinose, *trans*-caffeate and threonate, which were more abundant in the leaves, there was a significantly larger accumulation of metabolites in the spike organs compared to flag leaves (Figure 2). Additionally, the phenylpropanoids 3-cis and 3-trans-caffeoylquinic acids (CQAs), were only detected in the leaves. Concerning metabolomic differences between the two studied spike organs, many of the detected metabolites had accumulated to higher levels in the glumes than in the lemmas. Many amino acids (hydroxyproline, Hyp; proline, Pro; 5-oxoproline, ornithine, Orn; threonine, Thr; tryptophan, Trp and GABA) and sugars (fructose, Fru; glucose; Glc; sucrose, Suc; rhamnose, Rha and *myo*-inositol-P), as well as many metabolites involved in respiration and photorespiration (isocitrate; succinate; 3-phosphoglycerol; glycine, Gly; serine, Ser; glycolate and glycerate) as well as ascorbate metabolism (threonate, galactonate-1,4-lactone and glucarate-1,4-lactone) accumulated to significantly higher levels in glumes than in lemmas. In contrast, some sugars (erythrose, trehalose and maltose) and metabolites involved in respiration (2-oxoglutarate, 2OG; and pyruvate, Pyr) were more abundant in lemmas than in glumes.

Most of amino acids and all photorespiration intermediates decreased in all organs and conditions from anthesis to grain filling (Figure 2). Regarding major carbohydrates, Suc, Glc and Fru contents decreased in all organs from anthesis to the grain filling stage under WS whereas in HY conditions metabolic variation depended on the organ in particular. Lastly, trehalose, erythrose, fucose (Fuc) and maltose increased in leaves but decreased in spike organs from anthesis to grain filling. The relative contents of aromatic amino acids, Orn and threonate were higher during grain filling compared to anthesis in all organs and conditions and dehydroascorbic acid (DHA), histidine (His) and lysine (Lys) content in spike bracts were also increased at grain filling. With respect to respiratory metabolites, some were generally increased (isocitrate, citrate, malate) and other decreased (2OG, fumarate and Pyr) from anthesis to the grain filling stage and these changes were more pronounced under WS conditions.

### 3.2. Changes in the Metabolome Due to Water Stress

**Figure 3:**
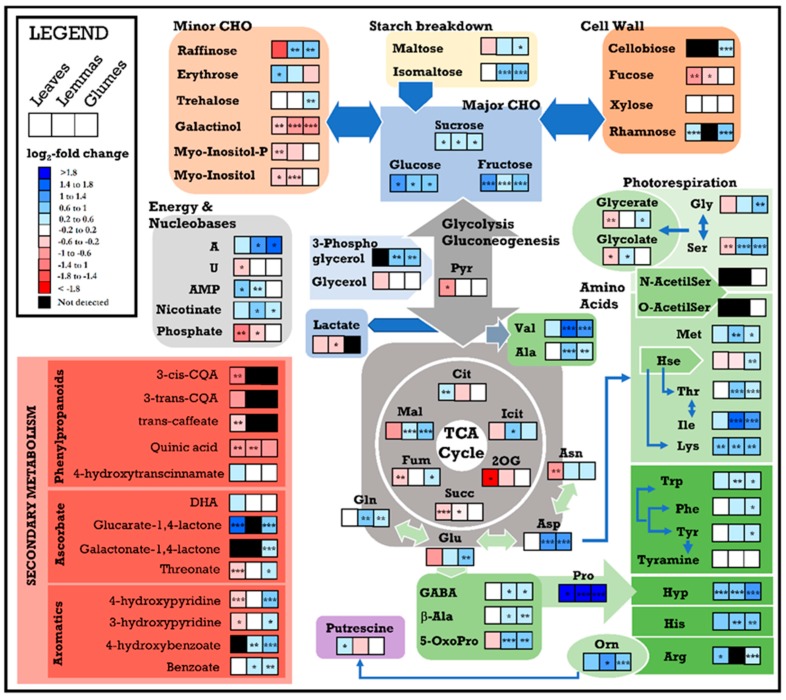
Water stress-induced changes in metabolite content at anthesis. Blue-red color scale represents log_2_-fold change, blue means higher metabolite content under water stress conditions and red stands for higher content under high-yielding conditions. Black indicates metabolites that were not detected. *, *p* < 0.05; **, *p* < 0.01; ***, *p* < 0.001.

Wide differences in the metabolomes of leaves and spike organs were found between WS and HY conditions, with these changes being partly dependent on the organ studied and phenological stage (Figure 3). In the following section, results are presented for the first sampling (anthesis) because at grain-filling, the differences in metabolite abundances between water conditions were less contrasted than at anthesis but the main trends were still comparable.

In all the studied organs, Fru, Glc and Suc contents increased significantly under WS compared to HY conditions. Similarly, raffinose, maltose, isomaltose and trehalose were generally increased in the spike organs, whereas erythrose increased in the flag leaves under WS. Galactinol decreased significantly in all the organs, particularly in the spikes, whereas myo-inositol and myo-inositol-P decreased in leaves and lemmas. Regarding cell wall metabolites, the Fuc relative content decreased in the flag leaves and lemmas, with Rha increasing in the leaves and glumes, whereas cellobiose increased only in glumes.

In the case of amino acids, WS generally induced an increase in all the studied organs, but this increase was particularly pronounced in the spikes. In both spike organs valine (Val), alanine (Ala), Asp, glutamine (Gln), methionine (Met), Thr, isoleucine (Ile), Lys, Trp, Hyp, GABA, β-Ala, 5-oxoproline, Pro, Orn and His increased in response to WS. Additionally, tyrosine (Tyr), phenylalanine (Phe), homoserine (Hse), glutamate (Glu) and arginine (Arg) content also increased in glumes under WS. In the leaves, Hyp, Arg, Lys and Pro increased significantly but asparagine (Asn) decreased under WS.

Whereas in leaves the relative content of photorespiratory metabolites decreased (glycerate, glycolate and Ser), in the spike bracts an increasing significant trend was observed under WS. In relation to the glycolytic pathway, Pyr decreased significantly in the leaves under WS. In the tricarboxylic acid (TCA) cycle, different changes were observed. In leaves 2OG, succinate and fumarate decreased, and citrate increased under WS. In the spike bracts malate increased under WS, whereas isocitrate increased and succinate decreased significantly in the lemmas alone, and fumarate decreased only in glumes. With regards to energy and nucleic acid metabolism, the nicotinate and adenine content increased significantly in glumes and lemmas. Meanwhile in leaves and lemmas, AMP increased and phosphate decreased under WS.

In addition, 3-phosphoglycerol increased in the spikes under WS whereas phenylpropanoids decreased significantly in the flag leaf (3-cis-caffeoilquinic acid, trans-caffeate and quinic acid) and in lemmas (quinic acid). The relative content of other secondary metabolites related to ascorbate metabolism (glucarate-1,4-lactone, galactonate-1,4-lactone and threonate) increased significantly in glumes but in leaves a variable trend was evidenced. Furthermore, in bracts there was a significant general increase across aromatic compounds.

### 3.3. Metabolic Differences between Genotypes with Contrasting Agronomic Performance

**Figure 4:**
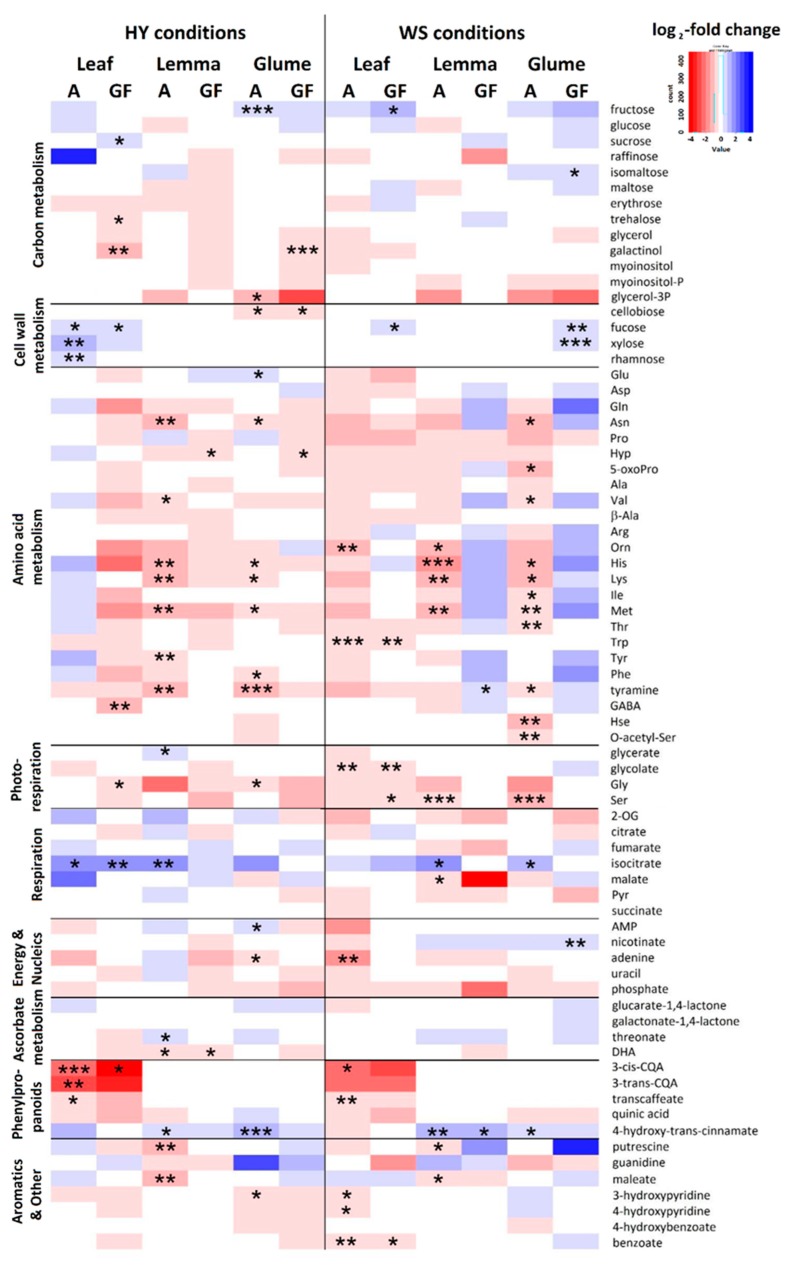
Genotypic differences in metabolite content between the drought-tolerant genotype Pelayo and the drought sensitive genotype Don Sebastian in leaves, lemmas and glumes at anthesis (S1) and grain filling (S2) under high-yielding (HY) and water stress (WS) conditions. The blue-red color scale represents log_2_-fold change, blue means higher metabolite content in Pelayo and red stands for higher content in Don Sebastian. Asterisks indicate significant genotypic differences. *, *p* < 0.05; **, *p* < 0.01; ***, *p* < 0.001.

Two genotypes contrasting in their performance under the two water conditions were considered: Pelayo, as a HY genotype, and Don Sebastian, as a low yielding (LY) genotype. These cultivars represented the extremes in terms of metabolite variation among the five cultivars used (Figure S2). The metabolite profiles of flag leaves, lemmas and glumes differed significantly between the two genotypes within each growing stage and environmental condition (Figure 4).

In leaves, Pelayo generally exhibited higher levels of major sugars, glycan-related sugars and others (Suc, Fru, Fuc, Rha, xylose and isocitrate) than in Don Sebastian, whereas the opposite occurred with phenylpropanoids, aromatics, some amino acids and other metabolites (CQAs, trans-caffeate, hydroxy-pyridines, adenine, GABA, Gly, Trp, Orn, Ser, glycolate, galactinol and trehalose). In the two spike bracts, Pelayo showed generally higher contents of 4-hydroxy-trans-cinnamate and isocitrate compared with Don Sebastian, while in lemmas there was higher contents of threonate and glycerate and in glumes higher levels of Fru, isomaltose, Fuc and xylose in Pelayo than in Don Sebastian.

At anthesis, spike bracts of Don Sebastian exhibited in general higher content in most of amino acids at anthesis and other metabolites such as DHA, malate and 3-phosphoglycerol than Don Sebastian. In contrast, the opposite trend was observed at grain filling but no significant differences between genotypes (except for Hyp in lemmas) were detected. It is likely that the higher amino acid contents in bracts of Don Sebastian at anthesis were due to lower rates of spike growth, while N assimilation continued to be active. In addition, the higher grain %N in Don Sebastian would likely be a consequence of the remobilization of N compounds to its lower number of grains spike^−1^ rather than a higher rate of N assimilation occurring in Don Sebastian compared to Pelayo.

### 3.4. Predicting Yield from Metabolite Profiles

The metabolome of the flag leaf blade and spike bracts proved strongly associated with GY and the closest fitting regression models were obtained when the metabolite profiles at anthesis were employed (Table 4). The metabolite profiles of leaves (raw metabolite intensity) explained up to 73.6% (Adj-R^2^) of GY variability in the training set and 65.2% of yield variability in the validation set (RMSE = 0.882). The metabolite profiles of the lemmas (log_2_-transformed metabolite intensity) explained up to 83.4% of yield variability in the training set and 65.5% in the validation set (RMSE = 0.878). Finally, the glume metabolite profiles (raw metabolite intensity) explained 78.4% of yield variability in the training set and 56.2% in the validation set (RMSE = 0.975). At the grain filling stage, the metabolite profiles of leaves, glumes and lemmas still explained much of the GY variability; 63.8%, 45.7% and 35.8% for the validation sets, respectively (Table 4).

The metabolites with the highest detection rate (DR) in the LASSO variable selection, along with their positive or negative effect on yield, are shown in Table 5. Calculation of the DR revealed variation in the importance of metabolite-GY associations between organs and growth stages. Whereas amino acids generally related to yield negatively, some organic acids and sugars related to protective, osmotic or cell wall metabolisms affected yield positively (Table 5). Thus, correlations of leaf and spike bracts metabolites with GY were particularly strong for fucose (positive correlation) and proline (negative correlation) (Figure S3).

**Figure 5:**
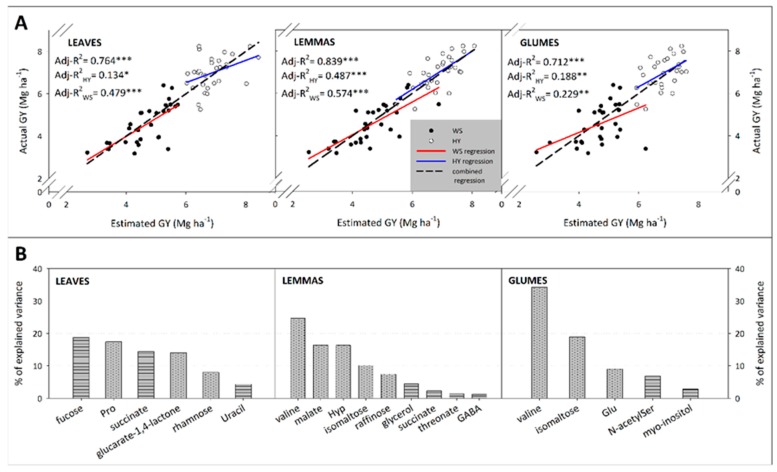
Multiple regression models with the variables selected by 70% of model runs (**A**) and their respective relative importance analyses for the variables (**B**). Blue: fitted line for high-yielding (HY) conditions, red: fitted line for water stress (WS), dotted line: fitted line for WS and HY conditions combined. Below, doted bars indicate metabolites negatively affecting grain yield (GY) and horizontal pattern bars indicate metabolites positively affecting GY.

In the multiple regression models (using metabolites with DR ≥ 70%), the metabolite profiles of leaves, lemmas and glumes significantly predicted yield variability in the whole set of data at anthesis as well as in the WS and HY growing conditions, separately (Figure 5A). In this analysis, the lemma metabolome provided the most accurate yield prediction from its metabolite profile, explaining up to 83.9% of yield variability, followed by leaves (Adj-R^2^ = 0.764) and glumes (Adj-R^2^ = 0.712) for the whole set of data. Despite all regressions being significant, under WS the metabolite variability always enhanced the yield prediction accuracy compared to HY conditions.

GY variance explained by the metabolites for the three organ-specific models showed that in leaves Fuc and Pro jointly explained above 36% of GY variation, succinate and glucarate-1,4-lactone explained about 28% and the remaining explained 12% of yield variation (Figure 5B). In bracts, Val explained between 25% to 34% and isomaltose between 10% to 19% of yield variation in lemmas and glumes, respectively. Besides, in lemmas malate and Hyp jointly reached 32% of yield variation, and raffinose and glycerol explained almost 12%, whereas succinate, threonate and GABA content jointly explained 5% of yield variation. Additionally, in glumes, the remaining Glu, N-acetylserine and *myo*-inositol jointly explained above 18% of yield variation.

At anthesis, the Pro (and usually Hyp) contents of the leaves, lemmas and glumes were associated with decreasing plant height (PH), biomass, HI, number of spikes m^−2^ and TKW and with increasing δ^13^C (Figure S4). Respiratory metabolites in both leaves and spike bracts were largely positively associated with increasing PH, biomass, HI, spikes m^−2^ and TKW. In leaves and glumes, the sugar alcohols (glycerol, galactinol and myo-inositol) were positively correlated with increasing HI, TKW, biomass, spikes m^-2^ and grains spike^−1^. However, 3-phosphoglycerol in bracts was negatively correlated with PH and HI and strongly positively correlated with δ^13^C. By contrast, amino acids in the leaves and bracts were generally positively associated with increasing spikes m^−2^, PH, HI and with decreasing grains spike^−1^ and biomass. Most amino acids also correlated negatively with δ^13^C, except Ala, Glu and β-Ala in the spikes, which correlated positively with this parameter. Several sugars (mostly maltose, isomaltose, Fru, and Glc, but in some cases Rha, trehalose, raffinose and erythrose) correlated negatively with spikes m^−2^, biomass, PH and HI, but positively correlated with grain δ^13^C. Fuc content in leaves and lemmas correlated positively with the number of grains m^−2^, GNY, HI, and Biomass.

## 4. Discussion

In this study, the decrease in biomass, GY and all yield components under WS conditions compared with the HY trials was associated with water stress as shown by the increase in leaf δ^13^C, an even larger increase in grain δ^13^C (Table 3) and the strong negative correlation between δ^13^C and grain yield across genotypes, plots and trials (adj.R^2^ = 0.405; *p* < 0.0001; not shown) [42,43]. In addition, thermal and spectral indices also evidenced the better water status in the HY trials at both the canopy and plant levels [44,45].

Genotypic differences in final yield among the five genotypes tested were partly explained by the decreased number of grains spike^−1^, which may be the consequence of smaller spike size together with a poorer grain set due to water stress [46].

### 4.1. Metabolic Overview of Wheat Flag Leaves and Spike Bracts and Their Phenology-Associated Changes

The observed higher levels of the majority of detected metabolites in glumes and lemmas compared to flag leaves (Figure 2) may be a consequence of i) the closeness to the grain [47] and ii) the active metabolic role of spikes as sink organs, but iii) also due to their role in assimilation and refixation [7,18]. Higher levels of the major sugars and of most of the amino acids in the spike bracts, particularly in the glumes, may indicate the significant contribution of wheat bracts to grain carbon, via photosynthesis and carbon refixation, and of nitrogen, via primary assimilation and/or recycling, particularly under water stress [7,16,17,18,19]. Higher levels of photorespiratory and TCA-cycle intermediates in the spikes suggest the occurrence of considerable photorespiratory rates, recycling of ammonia and provision of carbon skeletons which facilitated the increased synthesis and/or accumulation of amino acids in the spike bracts [48].

The ascorbate dependent detoxification machinery seemed to be increased in glumes, which is in agreement with recent work at the transcript and enzyme activity levels [7,14]. Furthermore, some sugars in lemmas (trehalose, maltose and erythrose) could have relevant functions as sugar storage, signaling and water stress tolerance [13,49,50,51]. In contrast, higher raffinose and malate content in leaves may evidence the accumulation of photosynthates [52] and/or a role in osmotic regulation [53] among other functions. Unlike spikes, the outstanding detection of phenylpropanoids in leaves may act as sun-screens and antioxidants [54,55].

Concerning metabolome changes from anthesis to grain filling (Figure 2), most metabolites decreased in leaves and spike bracts, which can be attributed to sugar and amino acid catabolism and remobilization to the grains. However, in spikes the aromatic and urea-cycle amino acids showed increasing trends over time, mainly under WS conditions, suggesting amino acid catabolism and N remobilization [56]. The increased intermediates of the TCA-cycle in the spikes, mainly under WS, could meet the increasing demand for amino and organic acids, via anaplerotic reactions, for their remobilization to the grain. Moreover, the increase in DHA and threonate levels in the spike bracts might suggest that antioxidant machinery, associated with these metabolites, could be involved in the maintenance of spike functioning at the end of the plant cycle [14].

### 4.2. Water Stress Effects on Flag Leaf and Spike Metabolomes

**Scheme 1:**
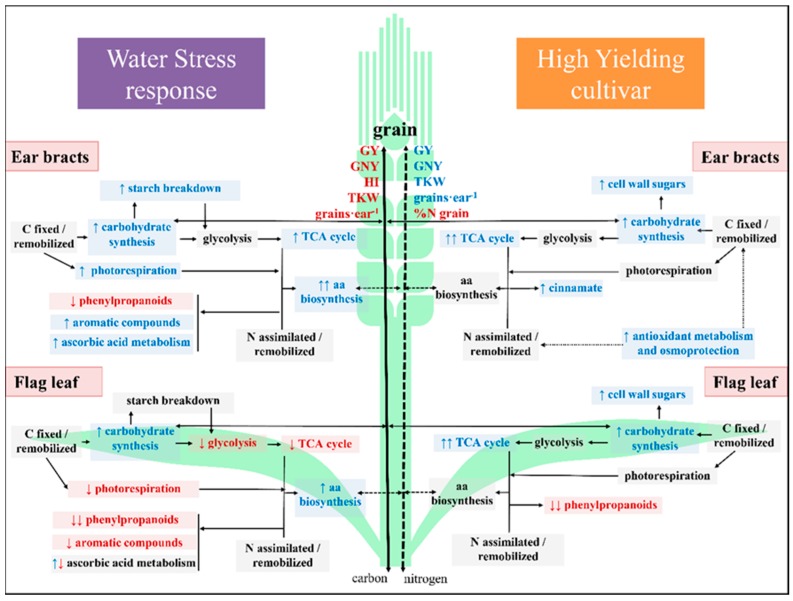
Illustration of the principal metabolic trends shown in leaves and spike bracts in response to water stress and in the high-yielding scenario. Blue and red colors refer to increasing and decreasing metabolic paths, respectively. Genotypic changes in yield components refer to the two-ways ANOVA by selecting the genotypes Pelayo and Don Sebastian only (data not shown). aa, amino acids; GNY, grain nitrogen yield; GY, grain yield; HI, harvest index; TKW, thousand kernel weight.

Water stress impacted broadly on leaf and spike bract metabolomes, particularly at the anthesis stage with a remarkable accumulation of major sugars and the amino acids Pro, Hyp and Lys (Figure 3, Scheme 1). These changes were likely due to growth cessation and a reduction in the consumption of metabolites and may participate in cell osmotic adjustment [57]. At grain filling, water stress-induced differences were mostly observed in the spike bracts (Table S3), which displayed a greater metabolic response than flag leaves [17,18,19].

The water stress-induced increase in amino acid content was remarkably more prominent in spike bracts than in flag leaves (Figure 3). In [7], an upregulation of *NR, NIR* and *GS2* genes, the key enzymes in the primary N assimilation pathway, was observed in durum wheat spikes, in contrast to flag leaves under water stress. These changes, together with the increase in several organic and amino acids in the present study, support an optimal coordination of carbon and nitrogen metabolism in spikes for the provision of nitrogen-rich compounds to the developing grain. This coordination was previously observed in durum wheat leaves [58], but has not previously been observed in the spikes.

In concordance with the changes observed in gene expression [7], respiratory intermediates generally decreased in leaves, while they increased in spike bracts under WS. The strong decrease in 2-oxogluatarate (a key metabolite required for ammonia assimilation) in leaves, could indicate a breakdown in the link between C and N metabolism under WS, while no changes were observed in spike bracts. At the same time, the increased photorespiration intermediates in spike organs in response to WS could be attributed to higher rates of photorespiration under drought [59]. This may support an enhancement of nitrogen recycling in coordination with respiratory metabolism, thereby contributing to the increased biosynthesis of amino acids in spike bracts [60,61]. In addition, the remarkable increase of the branched chain amino acids in spikes under WS may be physiologically relevant as alternative respiratory substrates under stress, either directly via electron transfer to the flavoprotein complex or indirectly via the TCA-cycle from their catabolic products [56,57].

Unlike leaves, the increase of other sugars in spikes, particularly in the glumes (raffinose, maltose, isomaltose and trehalose) may prevent oxidative damage [52] and could contribute to water stress tolerance [13,50] which partly explains the strong performance of spikes under stress [7,18]. Other water stress-induced metabolic changes concern structural elements (cell wall related sugars, phenylpropanoids and Hyp) perhaps promoting a modulation in the composition of the cell wall and membranes and cell wall thickening [60] assisting in tolerance of water stress.

Recent studies have reported higher antioxidant enzyme activities and transcript abundances in wheat spikes compared to flag leaves [7,14,62]. In our study, the increased precursors of ascorbate in glumes and leaves could be a consequence of a higher sun-exposition during the reproductive period of the crop. Complementarily, the moderate increase in aromatic amino acids in glumes under WS could be evidence of an increasing demand for precursors for the synthesis of antioxidants (e.g., flavonoids) [63]. The accumulation of other metabolites (GABA, 5-oxoproline, adenine and aromatics) in spike bracts under WS could also have a protective function [12,56,64,65].

Recent studies in wheat and other cereals have shown a remarkable upregulation of genes in the spike tissues that are involved in drought stress responses, including alternative oxidase, antioxidant system enzymes, heat shock proteins and dehydrins [7,66,67]. This upregulation likely leads to an improved capacity to counteract the accumulation of reactive oxygen species (ROS), particularly in the spike. On the other hand, the relevance of cysteine homeostasis (including cysteine as a sensitive target of ROS) and cysteine products (antioxidants and defense compounds) to prevent oxidative damage under osmotic stress has been demonstrated in plant species [68], including wheat [69]. Therefore, further research on cysteine metabolism in the spike in response to drought may provide valuable insights into the antioxidant system and its relationship with stress tolerance.

### 4.3. Metabolic Variation between Agronomically Contrasting Genotypes

Genotypic differences associated with yield performance (Figure 4, Scheme 1) involved two main metabolic events: changes in cell structural elements and carbon assimilation.

Firstly, in Pelayo (HY genotype) the accumulation of cell-wall monosaccharides, involved in glycan-structures synthesis, in the leaves and cinnamic acids, related with lignin composition, in the spikes suggest that alterations in the cell wall and membranes may occur contributing to drought acclimation and yield stability [70,71,72]. By contrast, the increase in leaf phenylpropanoids and precursors in Don Sebastian (LY genotype) may result in an increase in lignin deposition and consequently an inhibition of growth [73].

Secondly, increasing photosynthates in the leaves and glumes of the HY genotype suggest enhanced carbon assimilation including refixation [60] and carbon remobilization to the grain. This is in agreement with recent studies suggesting that the spike of Pelayo showed a greater capacity to re-fix CO_2_ respired by the grain [25,74]. The generally increased isocitrate content in leaves and bracts of Pelayo could be associated with energy metabolism, but also with secondary metabolism [75] and with the accumulation of carbon compounds when N is limited [76]. Further research on this topic may clarify how isocitrate contributes to genotypic outperformance.

Altogether, the difference in the metabolic traits between these two cultivars could partly explain the high stability of Pelayo and the poor adaptation of Don Sebastian to the growing conditions. In fact, recent studies have already illustrated the physiological and agronomical differences between both cultivars [25,74], including a preliminary assessment of the expression of stress-inducible and C/N metabolism genes [25]. Thus, compared to Don Sebastian, Pelayo had smaller and more erect flag leaves, exhibited better water status in terms of stomatal conductance, and had a better balance during grain filling between N sources and N sinks [25,74]. At the molecular level the better response of Pelayo was associated with higher transcription levels of genes involved in nitrogen (GS1 and GOGAT) and carbon (RCBL) metabolism, as well as water transport [25]. Moreover, in a detailed transcriptomic study currently in preparation, Vicente et al. compared the same cultivars and found a generalized upregulation of photosynthesis (ATPase, RBCL, RBCS, RCA, FBPase, SBPase) and nitrogen metabolism genes (NR, NIR, GS2, GS1, Fd-GOGAT, NADH-GOGAT), particularly nitrate reductase and nitrite reductase in Pelayo relative to Don Sebastian. In addition, genes encoding proteins involved in respiration and mitochondrial electron transport (PEPC(1), PEPC(2), PK, AOX) as well as the antioxidant system (CAT, SOD, GST-1, APX-1) were upregulated mainly in the spike tissues of Pelayo. Future validation of metabolic changes between the high-yielding cultivar, Pelayo, and the low-yielding cultivar, Don Sebastian, tested at the gene expression and protein level may clarify the molecular mechanisms that provide Pelayo with greater stability under Mediterranean agroclimatic conditions.

### 4.4. Prediction of Yield from Spike Bract and Flag Leaf Metabolomes

The LASSO statistical approach has proven its performance in metabolomic data sets with improvements over standard linear regression models, including PLS-based models [77]. Maize grain yield has been predicted satisfactorily from the leaf metabolome [24], revealing metabolic traits that may contribute to yield maintenance under abiotic stress conditions. In our study using wheat, the spike bract metabolome, particularly of the lemmas, proved to be as determinant for final crop yield determination as the leaves, confirming the relevance of spike metabolism [7,16].

The negative association of amino acids with yield (Table 5, Figure 5B) supports the concept that some metabolites classically assumed to have a drought-tolerance role (e.g., proline) [78] were instead stress indicators that probably accumulated as compatible solutes and osmoprotectants. Interestingly, Val rather than Pro seemed to play a key role in the spikes as indicators of stress severity and yield losses, and likely function as osmoprotectants and/or substrate for further energetic reactions [56,79]. Additionally, 3-phosphoglycerol in spike bracts appeared to be an excellent indicator of water stress as inferred by its strong correlation with grain δ^13^C (Figure S4), which increases with increasing water stress throughout crop growth.

In leaves, Fuc may confer firmer and more resistant cell walls [80], while succinate most likely feeds energetic and anaplerotic pathways, thus showed interesting metabolic targets for higher yields (Figures S3 and S4). In the spike bracts, HY performance was positively associated with, among other things (i) ascorbate and glutathione-related metabolites (*N*-acetylserine and threonate) which make up an essential antioxidant system and have a role in plant development and stress responses [64], (ii) myo-inositol as an abiotic stress tolerance inducer [24], (iii) GABA and glycerol likely involved in osmotic adjustment, redox control and carbon-nitrogen balance [81,82,83]. Therefore, this study suggests that the existence of active antioxidant and osmoprotective machinery in spike bracts is likely to have enabled appropriate functioning of other metabolic processes such as carbon and nitrogen assimilation during water stress conditions [62] or counteracted the typically higher temperatures observed in bracts compared to leaves [7], contributing positively to crop yield.

Finally, the positive correlation of leaf and spike bract respiratory intermediates and sugar alcohols with grain yield and most of the agronomical yield components (Figure S4), including HI and TKW, suggests a direct or indirect role of these metabolites in grain filling that should be studied further.

## 5. Conclusions

Our study revealed that the spike bract metabolome is strongly responsive to water stress, and far more noticeably than the flag leaf. Unlike leaves, a strong coordination between carbon and nitrogen metabolisms via primary nitrogen assimilation, the photorespiratory nitrogen cycle and the TCA-cycle was implicated in wheat spikes, particularly under WS, culminating in an active biosynthesis of organic acids and amino acids (Scheme 1). Additionally, the levels of carbon fixation and/or refixation in spikes might be substantial as inferred from the levels of photosynthates. The physiological outperformance of the spikes compared to the flag leaf under WS might be related to an active antioxidant machinery. Moreover, metabolite-GY association models revealed some metabolites potentially associated with genotypic outperformance, highlighting the association of respiratory, cell wall and antioxidant metabolites with water stress acclimation and yield stability. Drought resilience may be mediated, at least in part, by the high performance of the spikes. These findings therefore indicate that spike metabolic traits are a suitable breeding target. The results from the current study provide information relevant to the understanding of wheat physiology as well as providing potential biomarkers for yield prediction. Future work should enlarge the number and ideotype variability of the durum wheat varieties investigated, with the aim to confirm the value of these potential biomarkers.

## Figures and Tables

**Table 1:** Geographic, climatic, agronomic, and soil information for each study site. Colmenar de Oreja and El Majano experimental stations belong to the Instituto Nacional de Investigación y Tecnología Agraria y Alimentaria (INIA) of Spain and Zamadueñas experimental station belong to the Instituto Tecnológico Agrario de Castilla y León (ITACyL).

	Zamadueñas Experimental Station	Colmenar de Oreja Experimental Station	El Majano Experimental Station
Altitude (mamsl)	700	590	20
Coordinates	41° 42′ N, 4° 42′ W	40° 04′ N, 3° 31′ W	37° 14′ N, 6°03′ W
Mean Temp.^b^ (°C)	10.73	13.01	14.5
Max. mean Temp.^b^ (°C)	17.45	21.45	21.6
Min. mean Temp.^b^ (°C)	4.64	5.36	8.3
Precipitation ^b^ (mm)	258.4	206.8	161.8
Sowing date	24.11.2014	21.11.2014	11.12.2014
Harvest date	22.07.2015	20.07.2015	11.06.2015
Sowing density (seeds m^−2^)	250	250	250
Plot surface (m^2^)	10.5 (7 × 1.5)	10.5 (7 × 1.5)	10.5 (7 × 1.5)
Irrigation provided ^a^ (mm)	125	-	-
Fertilization			
1st application	300 kg ha^−1^ NPK 8:15:15	400 kg ha^−1^ NPK 15:15:15	500 kg ha^−1^ NPK 15:15:15
2nd application	300 kg ha^−1^ CAN 27%N	150 kg ha^−1^ Urea 46%	100 kg ha^−1^ Urea 46%
Soil texture	Loam	Clay-loam	Silty clay loam
Soil pH	8.44	8.1	7.6

^a^ in the irrigated treatment; ^b^ during the growing season.

**Table 2:** Means of grain yield (GY), grain nitrogen yield (GNY), biomass, thousand kernel weight (TKW), harvest index (HI), number of grains spike^−1^, grain and leaf nitrogen concentration for the high-yielding (HY) and water stress (WS) conditions and for each genotype along with the significance level of the respective two-way ANOVA. Values for conditions are averaged over genotypes and values for genotypes are averaged over conditions. Letters correspond to Tukey’s b separation. *p*-values are for conditions (P_C_), genotypes (P_G_) and the interaction (P_CxG_).

	GY (Mg ha^−1^)	GNY (kg ha^−1^)	Biomass (Mg ha^−1^)	HI (%)	TKW (g)	Grains Spike^−1^	Grain N (%)	Leaf N (%)
Conditions								
HY	6.98	165.3	19.53	36.04	49.48	34.62	2.39	3.92
WS	4.38	120.6	14.72	31.06	40.03	31.04	2.70	3.99
Genotypes								
Pelayo	6.26b	152.2	18.10	34.45ab	46.11b	33.05b	2.52ab	3.99
Kiko Nick	5.84ab	147.9	17.66	33.03ab	48.03b	28.01a	2.55ab	4.04
Dorondon	5.27ab	128.9	15.13	36.47b	38.77a	40.32c	2.38a	3.86
Sula	6.04b	138.0	17.83	33.64ab	40.74a	37.31c	2.50a	3.82
Don Sebastian	4.98a	135.5	17.40	30.64a	50.11b	25.45a	2.75b	4.01
Max.	8.24	203.5	35.14	43.15	63.80	51.10	3.48	4.83
Min.	3.18	71.7	9.45	17.10	27.90	21.40	0.98	2.71
CV (%)	25.9	21.6	24.3	17.9	17.3	21.8	14.5	9.4
ANOVA								
P_C_	0.000	0.000	0.000	0.002	0.000	0.002	0.000	0.375
P_G_	0.005	0.086	0.261	0.094	0.000	0.000	0.005	0.519
P_CxG_	0.063	0.517	0.716	0.935	0.918	0.166	0.104	0.788

**Table 3:** Means of canopy temperature (T), the canopy normalized difference moisture index (NDMI), the leaf normalized difference water index (NDWI) and the spike normalized water index (NWI) at the anthesis and grain filling stages and grain and leaf stable carbon isotope composition (δ^13^C) at grain filling under high-yielding (HY) and water stress (WS) conditions and for each genotype, along with the significance level of the respective two-way ANOVA. Values for conditions are averaged over genotypes and values for genotypes are averaged over conditions. Letters correspond to Tukey’s b separation. *p*-values are for conditions (P_C_), genotypes (P_G_) and the interaction (P_CxG_).

	Anthesis				Grain Filling				Mature Grains	
	T	Canopy NDMI	Leaf NDWI	Spike NWI	T	Canopy NDMI	Leaf NDWI	Spike NWI	Grain δ ^13^C (‰)	Leaf δ ^13^C (‰)
Conditions										
HY	15.71	−792	0.0442	−0.061	26.09	−695	0.0462	−0.061	−26.65	−28.47
WS	18.22	−747	0.0395	−0.068	33.44	−607	0.0394	−0.071	−25.03	−27.85
Genotypes										
Pelayo	17.40	−0.773	0.0402	−0.065 ab	29.96	−654	0.0412	−0.065	−26.00	−28.11
Kiko Nick	17.29	−0.761	0.0413	−0.068 a	31.53	−644	0.043	−0.074	−26.16	−28.50
Dorondon	17.61	−0.766	0.0414	−0.065 ab	30.77	−633	0.0401	−0.061	−26.04	−28.16
Sula	17.32	−0.782	0.0466	−0.067 a	31.16	−651	0.0478	−0.066	−26.00	−27.94
Don Sebastian	17.31	−0.767	0.0398	−0.058 b	31.53	−674	0.0422	−0.065	−25.49	−28.12
ANOVA										
P_C_	0.000	0.000	0.000	0.000	0.000	0.001	0.015	0.005	0.000	0.000
P_G_	0.952	0.683	0.058	0.007	0.952	0.885	0.145	0.150	0.699	0.196
P_CxG_	0.994	0.957	0.842	0.599	0.987	0.993	0.347	0.648	0.995	0.675

**Table 4:** Least absolute shrinkage and selection operator (LASSO) regression models for the prediction of grain yield from leaf, glume and lemma metabolite profiles (metabolites as predictor variables) at the anthesis and grain filling stages. Regression models were performed using raw metabolite intensity (upper part of table) and log_2_-transformed metabolite intensity. The statistics represent the mean across the 100 cross-validation runs.

	Anthesis Stage			Grain Filling Stage		
	R^2^	Adj R^2^	RMSE	R^2^	Adj R^2^	RMSE
Raw intensity						
Leaves						
Training set	0.801	0.736	0.758	0.774	0.702	0.805
Validation set	0.684	0.652	0.882	0.673	0.638	0.891
Glumes						
Training set	0.837	0.768	0.679	0.612	0.508	1.040
Validation set	0.602	0.562	0.975	0.437	0.381	1.180
Lemmas						
Training set	0.845	0.762	0.709	0.514	0.385	1.160
Validation set	0.651	0.616	0.925	0.252	0.178	1.370
Log_2_-transformed intensity						
Leaves						
Training set	0.855	0.788	0.669	0.808	0.741	0.744
Validation set	0.645	0.609	0.908	0.659	0.623	0.909
Glumes						
Training set	0.850	0.784	0.653	0.736	0.642	0.885
Validation set	0.582	0.539	0.998	0.507	0.457	1.110
Lemmas						
Training set	0.897	0.834	0.589	0.758	0.633	0.891
Validation set	0.687	0.655	0.878	0.417	0.358	1.210

R^2^, coefficient of determination; Adj-R^2^, adjusted coefficient of determination; RMSE, root mean square error.

**Table 5:** Ten metabolites at anthesis and grain filling showing the highest importance in yield prediction models based on 100 cross-validation runs and their effect on yield.

Leaves			Glumes			Lemmas		
Metabolite	Effect	DR (%)	Metabolite	Effect	DR (%)	Metabolite	Effect	DR (%)
Anthesis								
fucose	+	100	Val	−	93	Val	−	99
rhamnose	−	100	isomaltose	−	84	malate	−	98
Pro	−	99	Glu	−	83	Hyp	−	97
succinate	+	98	N-acetylSer	+	82	glycerol	+	96
glucarate-1,4-lactone	−	77	*myo*-inositol	+	77	threonate	+	79
uracil	+	73	cellobiose	−	69	GABA	+	75
galactonate	+	58	glycerol-3P	−	67	succinate	+	74
Trp	−	58	malate	−	66	raffinose	−	71
3-*cis*-caffeoylquinic acid	−	49	Asn	−	64	isomaltose	−	71
Asp	−	48	maltose	−	63	Ala	−	63
Grain filling								
fucose	+	100	fucose	+	100	trehalose	+	100
rhamnose	−	100	rhamnose	−	100	Asp	−	99
Trp	−	98	Trp	+	99	Hyp	−	98
phosphate	+	93	Glu	−	92	xylose	+	94
tyramine	−	88	Hyp	−	91	phosphate	+	85
Asn	+	87	Ala	+	66	citrate	−	84
β-Ala	−	79	salicylate	−	62	isocitrate	+	65
maltose	+	70	tyramine	+	53	4hydroxypyridine	+	64
Pro	−	68	xylose	+	49	succinate	−	63
erythrose	−	38	trehalose	+	44	4-hydroxy-*trans*-cinnamate	+	59

DR, detection rate in 100 cross validation runs; +/−, positive/negative effect of each metabolite on grain yield.

## Supplementary Materials

The below materials are available at https://www.mdpi.com/2073-4409/9/4/1025/s1. Table S1. Information on the cultivars tested; Table S2. Metabolite change in Zamadueñas; Table S3. Mean values of metabolite content; Figure S1. Yield variability; Figure S2. Metabolite variation among cultivars; Figure S3. Scatterplots of grain yield vs. metabolite content; Figure S4. Correlation network.
