# Supplementary material for: Metabolome Profiling Supports the Key Role of the Spike in Wheat Yield Performance
Source: Cells. 2020 Apr 21;9(4):1025. doi: 10.3390/cells9041025 (PMC7226616; doi:10.3390/cells9041025)
Supplement: Supplementary file 1 [file cells-09-01025-s001.pdf]

Metabolome Profiling Supports the Key Role of the Spike in Wheat Yield Performance

Supplementary Materials

Tables

Table S1. Information on the durum wheat cultivars tested. Year of release, country of registration, pedigree or cross name, breeder and use recommendation.

Cultivar	Year	Country	Pedigree/cross name	Breeder	Recommended use
Sula*	1994	Spain	Shearwater(SIB)/(SIB)Redneck//(SIB)Yavaros	CIMMYT	all cereal areas
Dorondon	1999	Spain	cross between Fs selected material	Genética y Gestión, S.C.	rainfed cold areas
Pelayo+	2003	Spain	Capeiti-8/Valnova (Eiti 6/Cappelli //Giorgio 324//Senatore Cappelli/Yuma)	Agrosa SA	cold areas
Don Sebastian*	2004	Spain	Zegzag-1/Lunde-5//Greenshank-32	Agrovegetal-CIMMYT	rainfed areas
Kiko Nick*	2009	Spain	SEL.CIMMYT-35/Durango//Isea-1938/Grazia	Limagrain Europe	all cereal areas

* bred either at CIMMYT or with a CIMMYT parent

+ Italian parents

Table S2. Metabolite content change (log₂-fold change) between the Zamadueñas rainfed and Zamadueñas irrigated trials at anthesis stage. Blue is indicative of increasing metabolite under WS environment. *, p-value < 0.05; **, p-value < 0.01; ***, p-value < 0.001; ns, non-significant.

	Leaves	Lemmas	Glumes
5-oxoproline	0.45**	0.51**	0.22ns
Ala	-0.41***	0.20*	0.36*
Arg	0.38**		0.18ns
Asn	-0.70*	-0.28ns	0.24ns
Asp	0.22ns	2.09***	1.89***
β-Ala	-0.34ns	0.21ns	0.39*
GABA	0.16ns	0.09ns	-0.05ns
Gln	0.22ns	0.60*	0.10ns
Glu	1.93**	1.15**	1.25**
His	0.31ns	0.57ns	0.05ns
Hse			0.32ns
Hyp	1.29***	1.00***	1.20***
Ile	-0.26ns	1.38***	0.85***
Lys	0.15ns	0.23ns	-0.03ns
Met	-0.22ns	0.60*	0.33ns
N-acetyl-Ser			-0.71***
O-acetyl-Ser			-0.54**
Orn	0.64ns	0.15ns	0.01ns
Phe	-1.56***	-0.08ns	-0.02ns
Pro	4.56***	3.09***	2.45***
Thr	0.02ns	0.73***	0.44***
Trp	-0.28ns	-0.31*	-0.19ns
Tyr	-1.44***	0.20ns	0.11ns
tyramine	-0.23ns	0.14ns	-0.06ns
Val	-0.06ns	1.47***	1.00***
3-hydroxypyridine	-0.33**	-0.09ns	0.59*
4-hydroxybenzoate		0.29ns	0.44ns
4-hydroxypyridine	-0.30*	0.00ns	0.35ns
benzoate	-0.35*	0.13ns	-0.02ns
lactate	-0.29ns	-0.22ns	
maleate	-0.29ns	0.12ns	0.11ns
putrescine	0.29ns	-0.20ns	-0.19ns
DHA	-0.13ns	0.27*	0.05ns
galactonate-1,4-lactone			0.55***
glucarate-1,4-lactone	1.43***		1.02***
threonate	-0.44***	-0.02ns	0.13ns
erythrose	0.46ns	0.69**	ns
Fru	1.02***	0.72***	0.88***
galactinol	-0.64***	-0.05ns	-0.19ns
Glc	1.18***	1.22***	1.22***

continued

	Leaves	Lemmas	Glumes
glycerol	-1.05***	-0.23*	-0.29**
glycerol-3P		1.26***	1.27***
isomaltose		0.82***	0.94***
maltose	0.28ns	0.52*	0.95ns
myo-inositol	-0.13ns	0.00ns	-0.02ns
*myo*inositol-1-P	-0.12ns	0.31ns	0.05ns
Raf	-0.74ns	0.51ns	0.75*
Suc	0.80***	1.04***	1.15***
trehalose	0.19ns	0.52***	0.90***
cellobiose			0.90***
fucose	-0.80***	-0.19*	0.01ns
rhamnose	0.25ns		0.43***
xylose	-0.34*	-0.32***	-0.24*
Adenine	-0.60ns	0.17ns	-0.56ns
AMP	0.76ns	0.75*	0.23ns
guanidine	-0.85ns	-0.55ns	-1.54ns
nicotinate	-0.47**	-0.14ns	-0.05ns
phosphate	0.03ns	-0.16ns	0.06ns
uracil	-1.15***	-0.16ns	-0.06ns
3-*cis*-CQA	-0.25ns		
3-*trans*-CQA	-0.14ns		
4-hydroxy-*trans*-cinnamate	0.31ns	0.27ns	0.00ns
quinic acid	-0.24ns		
trans-caffeate	-0.14ns		
Gly	-0.38*	0.38ns	0.52ns
glycerate	-0.33**	0.14ns	0.14ns
glycolate	-0.28*	0.02ns	0.20*
Ser	0.21ns	0.67***	0.79***
2OG	0.33ns	0.07ns	0.14ns
citrate		-2.04***	-2.43***
fumarate	-0.33ns	0.07ns	0.64**
isocitrate		-0.28ns	-0.33ns
malate	-0.37ns	0.79***	0.87*
pyruvate	-0.67**	0.34ns	0.01ns
succinate	-0.43***	-0.28*	-0.18ns

Table S3. Differences in the relative content of metabolites between growing conditions in leaves, lemmas and glumes at anthesis and grain filling expressed as log_2-fold change (log_2-FC) together with the corresponding statistical significance (p-value). Positive log_2-FC values indicate higher relative content under water stress conditions and negative log_2-FC values indicate higher content under high yielding conditions.

	Anthesis						Grain filling					
	Leaf		Lemma		Glume		Leaf		Lemma		Glume	
	log_2-FC	p-value	log_2-FC	p-value	log_2-FC	p-value	log_2-FC	p-value	log_2-FC	p-value	log_2-FC	p-value
Carbon metabolism												
Fru	1.31	0.000	0.56	0.000	0.95	0.000	0.35	0.053	0.48	0.006	0.55	0.028
Glc	1.37	0.000	0.85	0.000	0.84	0.000	0.17	0.307	0.54	0.002	0.59	0.034
Suc	0.36	0.016	0.37	0.011	0.39	0.014	0.12	0.631	0.09	0.506	0.23	0.064
raffinose	-1.68	0.164	0.65	0.003	0.58	0.008	0.12	0.591	0.50	0.351	0.15	0.549
isomaltose	-	-	0.80	0.000	0.88	0.000	-	-	0.05	0.673	0.04	0.835
maltose	-0.28	0.075	0.28	0.254	0.53	0.026	-0.33	0.026	-0.10	0.660	-0.10	0.573
glycerol	-0.25	0.225	0.14	0.220	0.06	0.553	-0.17	0.583	-0.13	0.491	-0.40	0.076
galactinol	-0.57	0.005	-0.66	0.000	-0.67	0.000	-0.09	0.553	-0.84	0.000	-0.39	0.007
trehalose	-0.05	0.072	0.19	0.114	0.50	0.002	-0.30	0.019	-0.76	0.000	-0.29	0.064
erythrose	0.76	0.046	0.56	0.148	-0.42	0.654	0.75	0.002	-0.08	0.816	-	-
myo-inositol	-0.21	0.016	-0.31	0.000	-0.14	0.068	0.08	0.448	0.21	0.381	0.02	0.922
*myo*inositol-1-P	-0.33	0.001	-0.25	0.132	-0.08	0.566	-	-	-0.11	0.461	-0.36	0.061
glycerol-3P	-	-	0.95	0.008	0.99	0.008	-	-	-	-	-0.38	0.526
Amino acid metabolism												
Hyp	0.80	0.001	0.65	0.000	1.07	0.000	0.47	0.066	0.57	0.003	0.52	0.037
Ala	-0.15	0.047	0.43	0.000	0.51	0.002	-0.07	0.669	-0.06	0.699	-0.06	0.570
Asn	-0.74	0.001	0.43	0.117	0.35	0.168	-0.26	0.409	0.26	0.612	0.17	0.690
Asp	-0.18	0.115	1.27	0.000	1.34	0.000	0.52	0.208	1.15	0.000	1.01	0.001
b-Ala	-0.08	0.512	0.25	0.038	0.39	0.004	0.59	0.001	0.31	0.059	0.07	0.648
Glu	-0.63	0.149	0.42	0.144	0.74	0.006	0.44	0.146	0.76	0.016	0.57	0.012
Gln	-0.20	0.437	0.81	0.005	0.44	0.007	-0.24	0.648	0.54	0.194	0.62	0.423
His	0.82	0.126	0.94	0.001	0.64	0.004	0.13	0.751	0.74	0.166	0.60	0.298
Ile	0.38	0.156	1.49	0.000	1.00	0.000	0.12	0.735	0.75	0.015	0.70	0.056
Lys	0.88	0.004	0.94	0.002	0.76	0.001	0.29	0.212	1.13	0.037	0.44	0.409
Met	0.44	0.276	0.69	0.001	0.55	0.004	-0.07	0.872	0.67	0.146	0.93	0.103
Orn	0.86	0.157	1.04	0.015	0.89	0.001	0.16	0.868	1.06	0.073	0.46	0.456
Phe	-0.13	0.630	0.21	0.139	0.43	0.035	0.11	0.772	0.06	0.824	0.27	0.527
Pro	2.98	0.028	2.96	0.000	2.47	0.000	1.33	0.069	1.27	0.005	1.19	0.011
5-oxoproline	-0.20	0.225	0.68	0.001	0.65	0.004	0.07	0.691	0.23	0.428	0.18	0.622
Thr	-0.14	0.104	0.83	0.000	0.51	0.000	0.16	0.344	0.49	0.016	0.40	0.052
Trp	0.22	0.187	0.33	0.006	0.31	0.012	0.42	0.005	-0.26	0.026	-0.25	0.045
tyramine	0.07	0.181	-0.04	0.941	-0.11	0.358	0.67	0.015	-0.01	0.938	-0.07	0.615
Tyr	-0.03	0.255	0.22	0.185	0.44	0.017	0.17	0.502	0.05	0.856	-0.03	0.934
Val	0.38	0.067	1.63	0.000	1.12	0.000	0.08	0.803	0.76	0.011	0.68	0.085
GABA	0.06	0.663	0.20	0.049	0.27	0.015	0.38	0.117	0.08	0.707	0.03	0.870
Arg	0.82	0.018	-	-	0.56	0.000	0.42	0.135	0.99	0.045	0.88	0.147
Hse	-	-	-	-	0.58	0.002	-	-	-	-	-	-

N-acetyl-Ser	-	-	-	-	0.07	0.616	-	-	-	-	-	-
O-acetyl-Ser	-	-	-	-	-0.05	0.815	-	-	-	-	-	-
Photorespiration												
glycerate	-0.54	0.003	0.10	0.196	0.20	0.014	-0.38	0.154	0.06	0.770	-0.10	0.502
glycolate	-0.21	0.026	0.18	0.022	0.12	0.118	0.04	0.838	0.04	0.748	-0.14	0.461
Gly	-0.34	0.121	0.37	0.533	0.84	0.006	-0.44	0.030	0.44	0.055	0.33	0.263
Ser	-0.43	0.002	0.78	0.000	0.78	0.000	-0.04	0.863	0.36	0.181	0.33	0.249
Respiration												
2OG	-2.56	0.015	-0.48	0.077	-0.08	0.789	-0.74	0.543	0.01	0.915	0.75	0.098
citrate	0.45	0.003	-0.32	0.316	0.00	0.990	-0.18	0.459	0.32	0.144	0.42	0.034
fumarate	-0.30	0.005	-0.15	0.398	0.38	0.017	0.08	0.696	0.33	0.260	0.33	0.204
isocitrate	-0.33	0.478	0.71	0.012	0.52	0.055	-0.22	0.209	0.35	0.163	0.14	0.492
MALATE	-0.88	0.145	0.59	0.000	0.99	0.000	0.00	0.979	1.15	0.320	-0.94	0.007
pyruvate	-0.92	0.023	0.14	0.508	-0.02	0.868	-0.44	0.050	0.26	0.563	0.53	0.230
succinate	-0.54	0.000	-0.18	0.040	0.08	0.409	-0.14	0.339	0.05	0.815	-0.03	0.866
Cell Wall												
cellobiose	-	-	-	-	0.50	0.000	-	-	-	-	-0.05	0.859
fucose	-0.65	0.001	-0.35	0.003	-0.06	0.446	-0.45	0.000	-	-	-0.68	0.000
xylose	0.00	0.994	-0.08	0.571	0.02	0.893	-0.14	0.366	-0.02	0.848	-0.21	0.022
rhamnose	0.45	0.000	-	-	0.61	0.000	0.42	0.000	-	-	0.13	0.269
Energy & Nucleic												
AMP	0.86	0.037	0.58	0.005	0.15	0.270	-	-	-	-	0.14	0.630
nicotinate	0.34	0.064	0.65	0.027	0.41	0.033	0.02	0.897	0.16	0.344	-0.22	0.044
guanidine	-1.87	0.137	0.37	0.266	-1.12	0.340	0.64	0.156	0.23	0.353	0.05	1.000
Adenine	0.22	0.623	1.06	0.019	1.41	0.032	-	-	0.01	0.992	-	-
uracil	-0.48	0.019	0.16	0.263	0.15	0.314	0.23	0.447	0.80	0.004	0.09	0.758
phosphate	-1.20	0.002	-0.49	0.039	-0.19	0.372	-0.72	0.028	-0.11	0.842	-0.69	0.078
Ascorbate metabolism												
DHA	0.22	0.124	0.10	0.414	-0.02	0.617	0.17	0.314	0.07	0.633	0.19	0.163
glucarate-1,4-lactone	1.53	0.000	-	-	0.74	0.000	-	-	-	-	0.81	0.025
galactonate-1,4-lactone	-	-	-	-	0.38	0.000	-	-	-	-	0.04	0.759
threonate	-0.30	0.000	0.19	0.062	0.29	0.001	0.04	0.704	0.02	0.907	-0.11	0.227
Phenylpropanoids												
4-hydroxy-*trans*-cinnamate	0.51	0.138	0.14	0.340	0.12	0.432	0.02	0.943	-0.16	0.397	-0.36	0.067
3-*cis*-CQA	-1.03	0.007	-	-	-	-	0.07	0.966	-	-	-	-
3-*trans*-CQA	-0.80	0.088	-	-	-	-	-0.16	0.811	-	-	-	-
trans-caffeate	-0.31	0.008	-	-	-	-	-0.02	0.808	-	-	-	-
quinic acid	-0.95	0.001	-0.90	0.002	-0.62	0.101	-0.74	0.026	-0.33	0.157	-0.85	0.002
Aromatics & Other												
4-hydroxybenzoate	-	-	0.41	0.006	0.72	0.001	-	-	0.26	0.103	-0.04	0.874
benzoate	-0.12	0.202	0.20	0.033	0.26	0.006	0.09	0.498	0.14	0.259	-0.40	0.018
3-hydroxypyridine	-0.39	0.000	0.13	0.347	0.60	0.000	-0.20	0.237	-	-	-0.13	0.404
4-hydroxypyridine	-0.39	0.034	0.03	0.819	0.29	0.037	-0.24	0.214	-0.08	0.544	-0.10	0.627
lactate	-0.33	0.059	-0.23	0.032	-	-	-0.28	0.092	-0.50	0.119	-0.25	0.468
malonate	-	-	-	-	-	-	-	-	-	-	-0.93	0.000
salicylate	-	-	-	-	-	-	0.07	0.609	-	-	-0.09	0.160
putrescine	0.46	0.013	-0.28	0.110	-0.14	0.513	0.09	0.711	-	-	0.84	0.340
maleate	-0.22	0.512	0.33	0.094	3.92	0.482	0.16	0.590	0.33	0.260	-0.94	0.014

Figures

Figure S1. Mean values of grain yield in a collection of twenty post-green revolution durum wheat genotypes under high yielding (HY) and water stress (WS) conditions as indicated in the Materials and Methods. Red bars correspond to the five selected genotypes in this study.

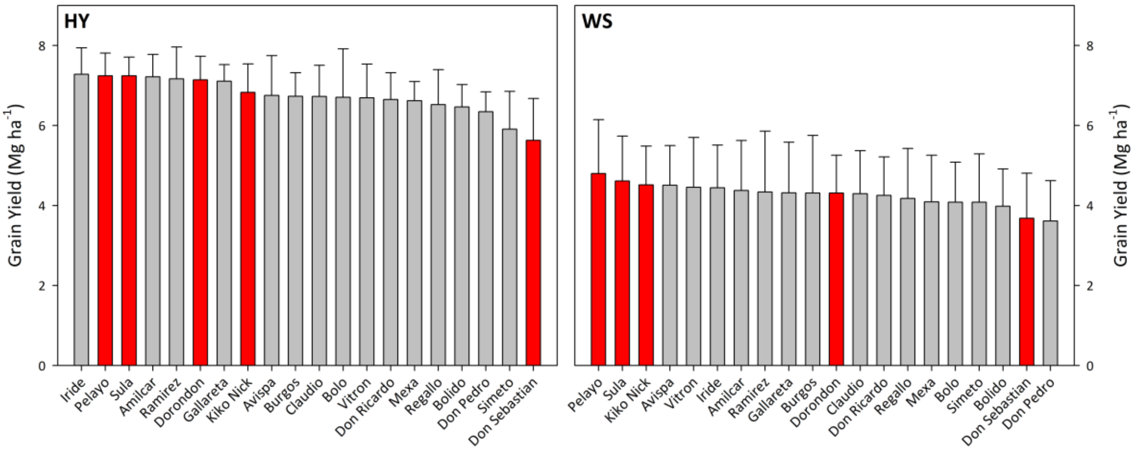

Figure S2. Heatmaps of metabolite profiles of leaves, lemmas and glumes at anthesis under high yielding (HY) and water stress (WS) conditions for each of the five tested cultivars: Pelayo, PE; Don Sebastian, DS; Dorondon, DO; Sula, SU and Kiko Nick, KN. The red-blue color scale was obtained from Z-score transformation of actual values. Grey indicates metabolites that were not detected.

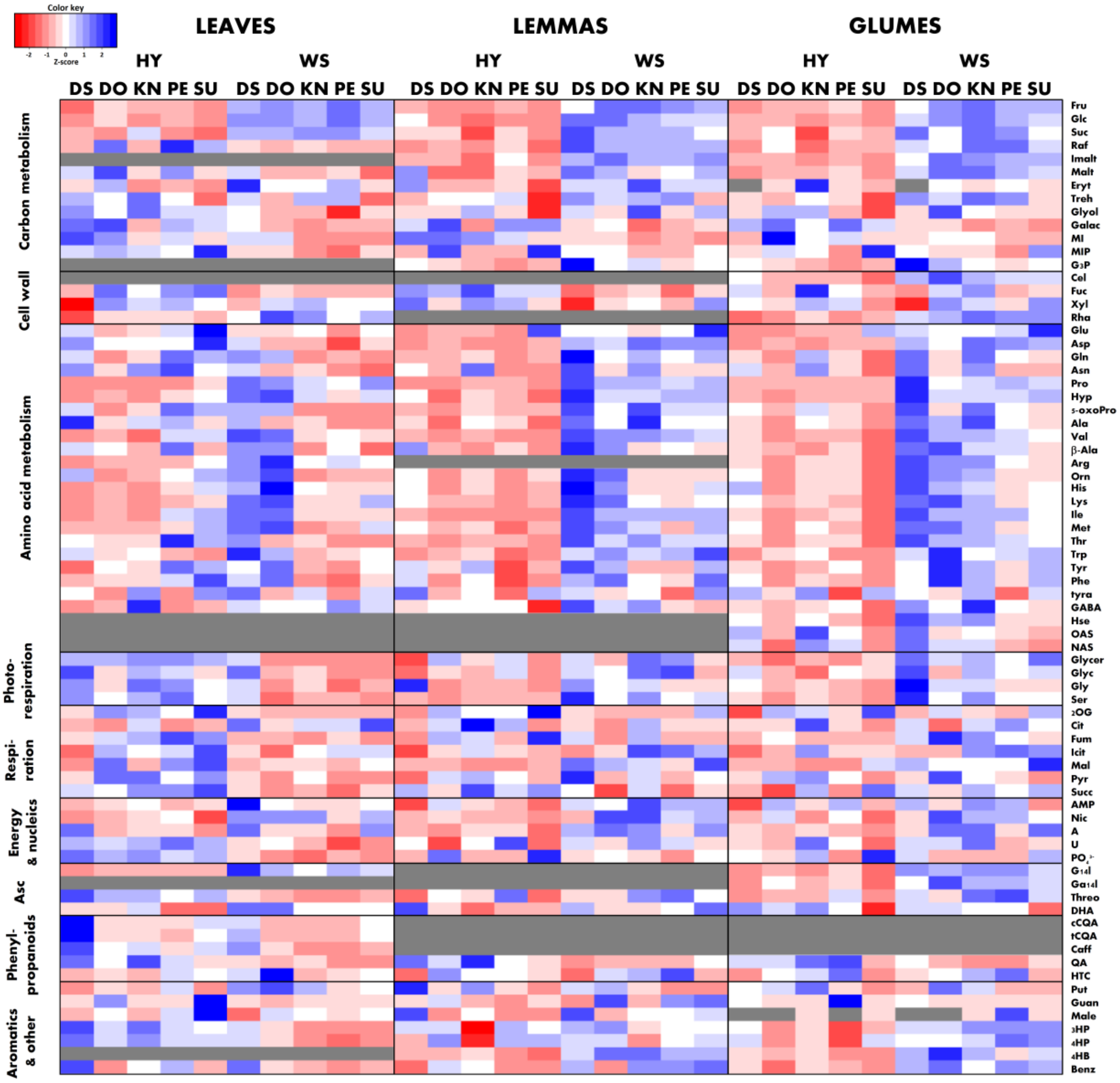

2OG, 2-oxoglutarate; cCQA, 3-cis-caffeoylquinic acid; 3HP, 3-hydroxypyridine; G3P, 3-phosphoglycerol; tCQA, 3-trans-caffeoylquinic acid; 4HB, 4-hydroxybenzoate; Hyp, 4-hydroxyproline; 4HP, 4-hydroxypyridine; 4HP, 4-hydroxypyridine; HTC, 4-hydroxy-trans-cinnamate; 5-oxoPro, 5-oxoproline; A,adenine; AMP, adenosine monophosphate; Ala, alanine; Arg, arginine; Asn, asparagine; Asp, aspartate; β-Ala, β-alanine; Benz, benzoate; BA, benzylalcohol; Cel, cellobiose; Cit, citrate; DHA, dehydroascorbate; Eryt, erythrose; Fru, fructose; Fuc, fucose; Fum, fumarate; Galac, galactinol; Ga14l, galactonate-1,4-lactone; GABA, γ-aminobutyric acid; Gl14l, glucarate-1,4-lactone; Glc, glucose; Glu, glutamate; Gln, glutamine; Glycer, glycerate; Glyol, glycerol; Gly, glycine; Glyc, glycolate; Guan, guanidine; His, histidine; Hse, Homoserine; Icit, isocitrate; Ile, isoleucine; Imalt, isomaltose; Lys, lysine; Mal, malate; Male, maleate; Malt, maltose; Met, methionine; MI, myo-inositol; MIP, myoinositol-1-P; NAS, N-acetyl-Serine; Nic, nicotinate; OAS, O-acetyl-Serine; Orn, ornithine; Phe, phenylalanine; phosphate, PO_4^{3-}; Pro, proline; Put, putrescine; Pyr, pyruvate; QA, quinic acid; Raf, raffinose; Rha, rhamnose; Ser, serine; Succ, succinate; Suc, sucrose; Threo, threonate; Thr, threonine; Caff, trans-caffeate; Treh, trehalose; Trp, tryptophan; Tyra, tyramine; Tyr, tyrosine; U, uracil; Val, valine; Xyl, xylose.

Figure S3. Scatter plots between grain yield and fucose (Fuc), proline (Pro) and hydroxyproline (Hyp) relative content in leaves, glumes or lemmas at anthesis and grainfilling stages. *, p-value < 0.05; **, p-value < 0.01; ***, p-value < 0.001; ns, non-significant.

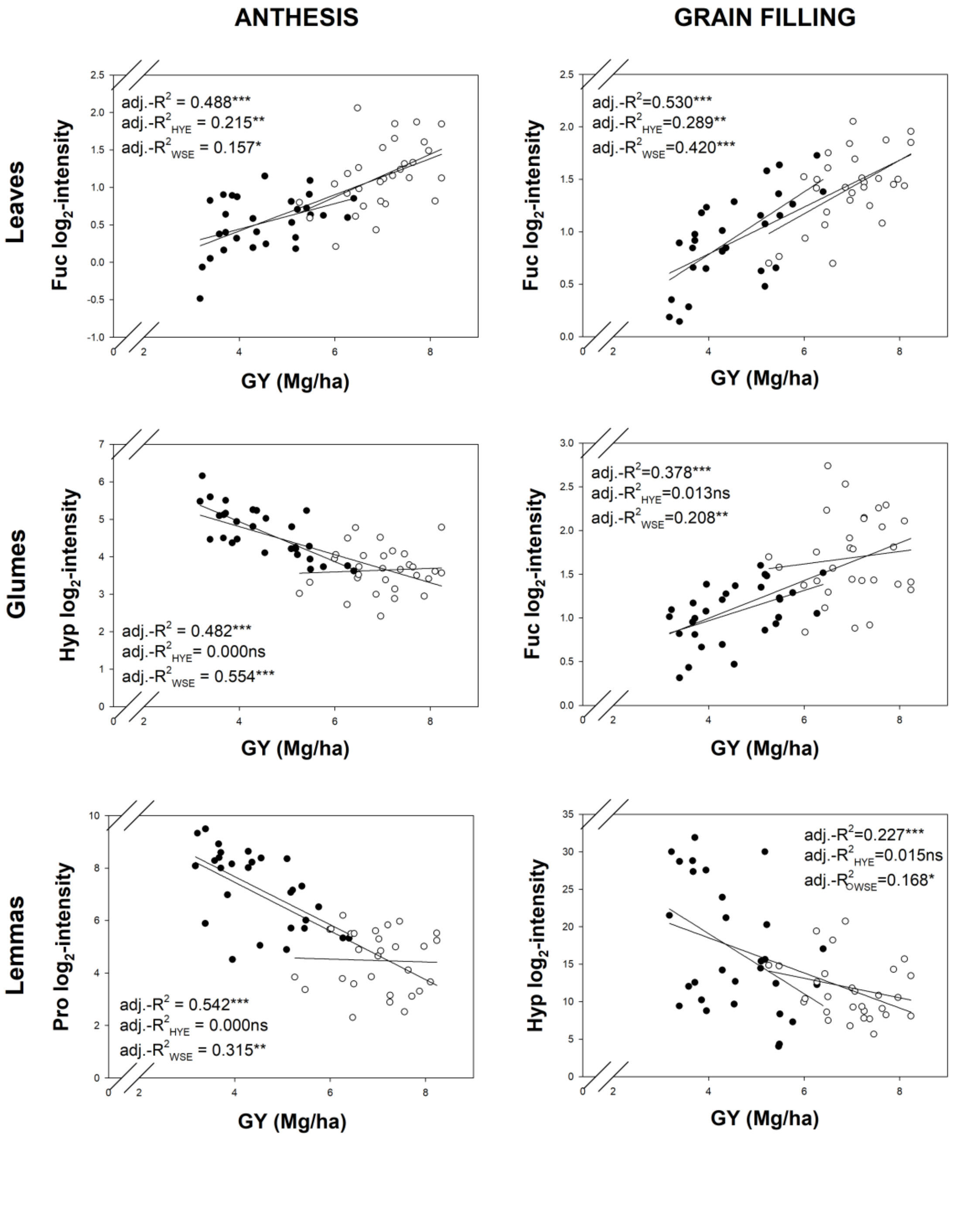

Figure S4. Correlation networks between metabolite contents and agronomic and physiological traits. The maximum alpha value was fixed at 0.001 and weaker correlations were omitted. Green lines stand for positive correlations and red lines for negative correlations. Greater line thickness indicates higher correlation coefficients.

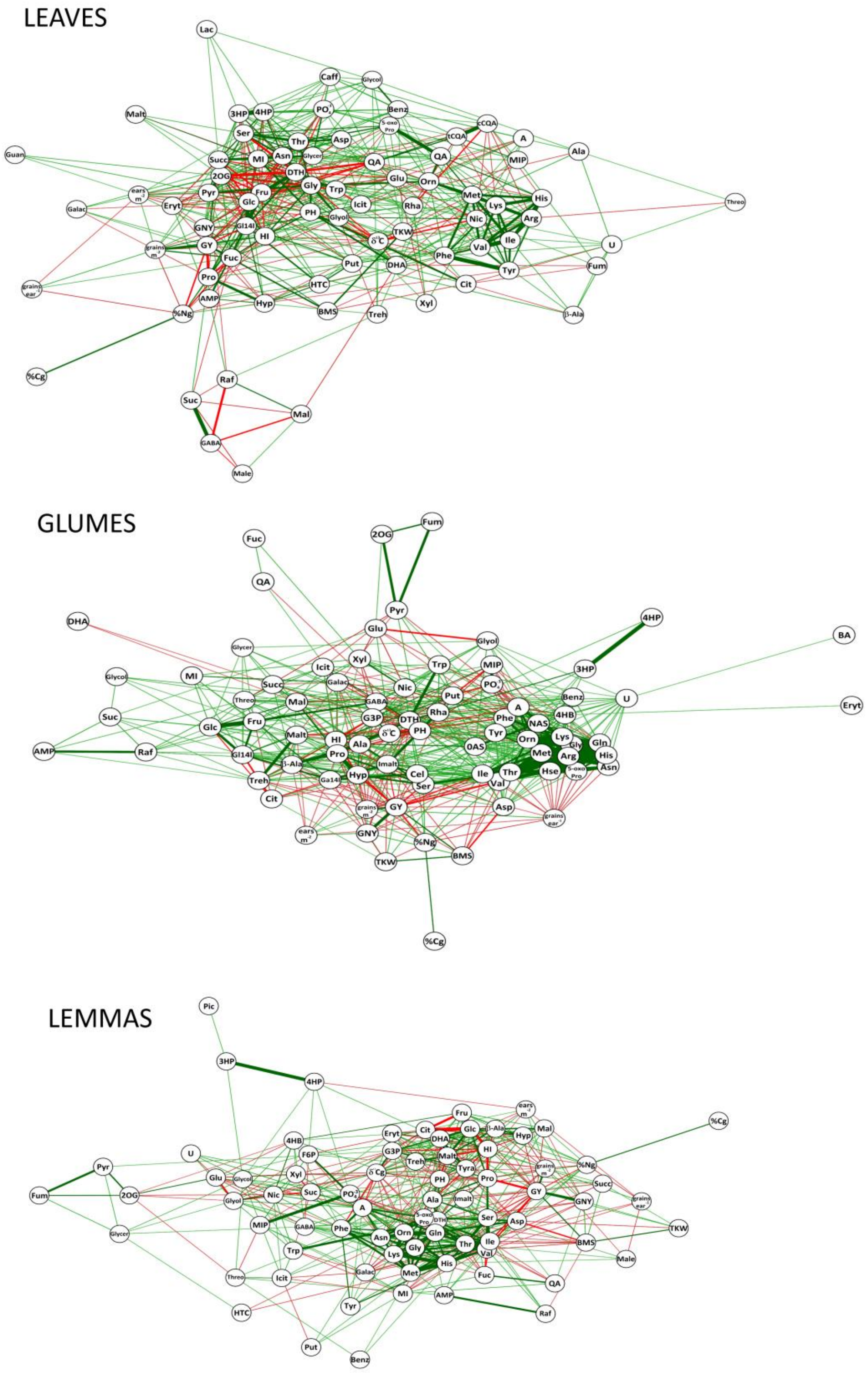

2OG, 2-oxoglutarate; cCQA, 3-cis-caffeoylquinic acid; 3HP, 3-hydroxypyridine; G3P, 3-phosphoglycerol; tCQA, 3-trans-caffeoylquinic acid; 4HB, 4-hydroxybenzoate; Hyp, 4-hydroxyproline; 4HP, 4-hydroxypyridine; 4HP, 4-hydroxypyridine; HTC, 4-hydroxy-trans-cinnamate; 5-oxoPro, 5-oxoproline; A, adenine; AMP, adenosine monophosphate; Ala, alanine; Arg, arginine; Asn, asparagine; Asp, aspartate; β-Ala, β-alanine; Benz, benzoate; BA, benzylalcohol; BMS, biomass; $\delta^{13}C$, carbon isotope composition; Cel, cellobiose; Cit, citrate; DTH, days to heading; DHA, dehydroascorbate; ears m^{-2}, ears per square meter; Eryt, erythrose; Fru, fructose; F6P, fructose-6-phosphate; Fuc, fucose; Fum, fumarate; Galac, galactinol; Ga14l, galactonate-1,4-lactone; GABA, γ-aminobutyric acid; Gl14l, glucarate-1,4-lactone; Glc, glucose; Glu, glutamate; Gln, glutamine; Glycer, glycerate; Glyol, glycerol; Gly, glycine; Glycol, glycolate; %Cg, grain carbon concentration; %Ng, grain nitrogen concentration; GNY, grain nitrogen yield; GY, grain yield; grains ear^{-1}, grains per ear; grains m^{-2}, grains per square meter; Guan, guanidine; HI, harvest index; His, histidine; Hse, Homoserine; Icit, isocitrate; Ile, isoleucine; Imalt, isomaltose; Lac, lactate; Lys, lysine; Mal, malate; Male, maleate; Malt, maltose; Met, methionine; MI, myo-inositol; MIP, myoinositol-1-P; NAS, N-acetyl-Serine; Nic, nicotinate; OAS, O-acetyl-Serine; Orn, ornithine; Phe, phenylalanine; phosphate, PO_4^{3-}; Pic, picolinate; PH, plant height; Pro, proline; Put, putrescine; Pyr, pyruvate; QA, quinic acid; Raf, raffinose; Rha, rhamnose; Ser, serine; Succ, succinate; Suc, sucrose; TKW, thousand kernel weight; Threo, threonate; Thr, threonine; Caff, trans-caffeate; Treh, trehalose; Trp, tryptophan; Tyra, tyramine; Tyr, tyrosine; U, uracil; Val, valine; Xyl, xylose.
